# Caffeine as a Gelator

**DOI:** 10.3390/gels2010009

**Published:** 2016-03-02

**Authors:** Nonappa, Erkki Kolehmainen

**Affiliations:** 1 Department of Chemistry, University of Jyväskylä, Jyväskylä FI-40014, Finland; erkki.t.kolehmainen@jyu.fi; 2 Department of Applied Physics, Aalto University School of Science, Puumiehenkuja 2, Espoo FI-02150, Finland

**Keywords:** caffeine, gels, supramolecular chemistry, solid state NMR

## Abstract

Caffeine (a stimulant) and ethanol (a depressant) may have opposite effects in our body, but under *in vitro* conditions they can “gel” together. Caffeine, being one of the widely used stimulants, continued to surprise the scientific community with its unprecedented biological, medicinal and physicochemical properties. Here, we disclose the supramolecular self-assembly of anhydrous caffeine in a series of alcoholic and aromatic solvents, rendering a highly entangled microcrystalline network facilitating the encapsulation of the solvents as illustrated using direct imaging, microscopy analysis and NMR studies.

## 1. Introduction

Coffee, with its flavor, aroma and stimulant properties, is one of the extensively consumed beverages in the human history [1]. The stimulant properties of coffee and tea are attributed to the presence of caffeine, which represents one of the widely used alkaloids [2]. The presence of caffeine is not restricted to coffee beans and tea leaves, but found in a number of other plants and plant products [3,4,5,6,7]. In spite of having several centuries of history, numerous biological, medicinal, physicochemical, as well as materials properties of caffeine have remained undisclosed and continued to surprise the scientific community [8,9,10]. Recently, caffeine has gained importance in genetics as an elegant example for convergent evolution [11]. The biological importance of caffeine as an anticancer agent, in prevention of Alzheimer disease, controlling the amyloid fibril formation and as a potential neuroactive drug has attracted the modern research community [12,13,14,15]. In supramolecular chemistry, the studies dealing with solid-state structural properties of caffeine and its derivatives continued to emerge rapidly [16,17,18]. The polymorphic forms, solvates, co-crystals and inclusion complexes of caffeine have been well documented in the literature [19,20,21]. The first X-ray diffraction studies by June Sutor showed that caffeine readily crystallizes in its monohydrate form [22]. The monohydrate form readily undergoes a transformation under ambient conditions into an anhydrous β-form. However, at higher temperatures the β-form undergoes phase transition into anhydrous α-form [23]. During this transition process, the single crystals are often destroyed, making the structure determination a challenging task. Therefore, the X-ray crystal structure determination of pure anhydrous form of caffeine remained a mystery until recently. In 2007, the first successful crystal structure was reported for anhydrous caffeine by Lehman *et al*. [24]. Using X-ray powder diffraction and high resolution solid state NMR, Enright and co-workers studied the anhydrous forms of caffeine [25]. Eddleston *et al.* showed that caffeine undergoes crystallization into tubular microcrystals in a wide range of solvents [26]. A vast number of crystallization of caffeine were carried out using solvent evaporation method. However, the self-assembly of caffeine leading to immobilization of solvent has not been reported in the literature.

Self-assembled supramolecular gels derived from low molecular weight organic gelators (LMOG) are attractive building blocks for drug delivery, tissue engineering and nanoscience [27,28,29]. The tremendous interest in this field over the past two decades is attributed to the ability of small molecules to generate micro- and nanofibers, tapes or rod-like self-assembled superstructures, which trap and immobilize a wide variety of solvents [30,31,32,33]. This property of small molecules offers a unique opportunity towards the careful and systematic design of gelators with tunable material properties [34,35]. The gelation ability of peptides, steroidal derivatives, aromatic charge transfer complexes, supramolecular metallogelators, have been well documented in the literature [27,28,29,30,31,32,33,34,35,36,37,38,39]. The morphological details have been studied using TEM, Cryo-TEM, environmental SEM, AFM and optical microscope [40,41,42,43]. Several non-invasive methods such as fluorescence spectroscopy, UV-Vis, circular dichroism, solution NMR, as well as Solid state NMR, have been utilized to study gels in their native state [44,45,46,47,48]. One of the hypotheses that, the gelation is due to 3D fibrillar network established via supramolecular self-assembly of small molecules. The size of the network varies significantly from one gelator to another as well as from one solvent to another for a given gelator. When the gel networks are at nanometer regime, it poses a challenge to visualize and provide a direct evidence towards 3D network formation leading to gelation in its native state. We point out that, the microscopy studies require a different imaging environment, or surface to study the self-assembly compared to a native gel, as a result the growth of fibers at its native gelation environment is limited. Here, we show that an anhydrous form of caffeine undergo supramolecular self-assembly leading to microcrystalline network and immobilize the solvents at a concentration as low as 1.0 w/v%. The microcrystalline (*d* = >2 µm) network allows the direct visualization of the evolution and solvent encapsulation during gelation in its native state.

## 2. Results and Discussion

**Figure 1:**
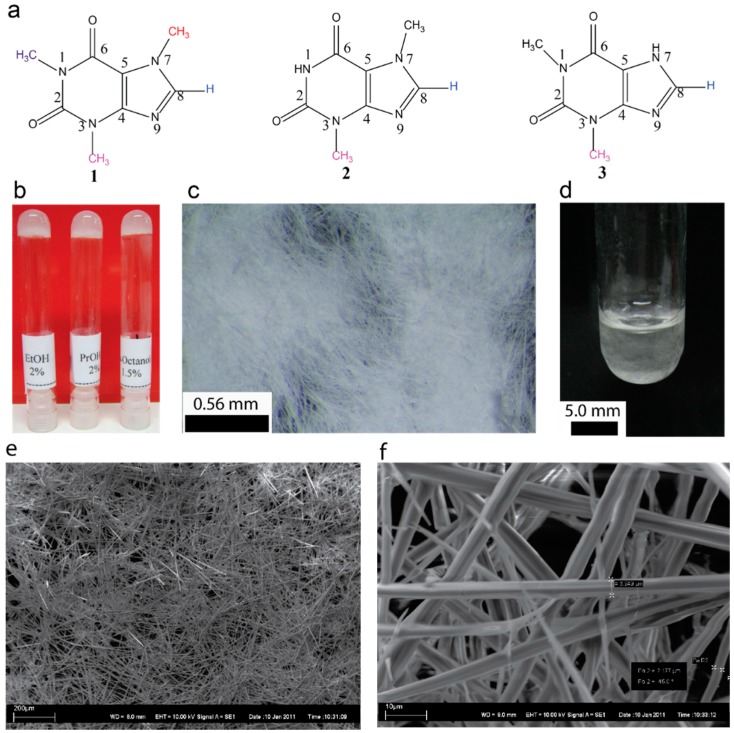
(**a**) Chemical structures of molecules used in this study, caffeine **1**, theobromine **2**, and theophylline **3**; (**b**) representative photographs of gels (2.0 w/v%) derived from caffeine; (**c**) 1-propanol gel upon placing on a glass slide shows entangled microfibrillar network; (**d**) photograph of 2.0 w/v% of caffeine in 1-propanol upon cooling a hot solution shows the fiber network before forming a stable gel; (**e**,**f**) SEM micrographs of 1-Octanol xerogel (2.0%).

Caffeine (Figure 1a) being one of widely used alkaloids, can either be extracted from various sources or obtained commercially [34,35]. Caffeine is readily soluble in water (20 g/L at room temperature and 660 g/L at boiling temperature). The solubility in ethanol is relatively high (15 g/L). However, in our study when the commercial caffeine was recrystallized from ethanol, it resulted in an anhydrous form of caffeine. Surprisingly, when the recrystallized caffeine (see experimental Secion 4.2 for details) was dissolved in ethanol (2.0 w/v%) by heating and allowed to attain room temperature, the solution turned into a gel (Figure 1) and showed the resistance to flow upon inversion. More importantly, this property of caffeine has never been reported in the literature. Similar studies using other xanthines such as theobromine and theophylline showed that the gelation is unique to caffeine (Figure 1a).

Inspired by this observation, we undertook a systematic study on recrystallized form of caffeine. The gelation was observed in a number of alcohols with minimum gelation concentration of 1.0 w/v%. We point out that the aggregation of small molecules above 2.0% leading to gel like structure is common, therefore we do not include any solvent where the gels are formed above 2.0%. The gelation properties of caffeine was studied in 15 different alcoholic solvents and aromatic solvents (see Supplementary Materials, Table S1 for details).

We observed that the gels obtained from alcoholic solvents are opaque or translucent depending on the chain length (Figure 1b). The gelation occurred within 10–30 min upon allowing a hot solution containing a known amount of caffeine to attain the room temperature. Gelation at 1.0% required mild sonication of the hot solution for one minute. On the other hand, for 2.0% gels in alcoholic solvents, no sonication was required. Similarly, aromatic solvents such as chlorobenzene and toluene furnished a gelatinous fluid upon attaining the room temperature. However, sonicating the hot solutions for 1.0 min stable gels were obtained. Typical macroscopic and microscopic appearances of the selected organogels are shown in Figure 1b–f (see supplementary Figures S1 and S2). The gel melting temperature increased with increasing the chain length of alcohols (see Supplementary Figure S3). For a given solvent the gel melting temperature increased linearly with increasing the concentration (see Supplementary Figure S4).

The gels remained stable for several months and placing the gel over a glass surface shows the presence of high aspect ratio microcrystalline network (Figure 1c). Interestingly, the formation of crystalline network when a hot solution was allowed cool can be visualized directly in a test tube used for gelation studies as shown in Figure 1d. The scanning electron micrographs of xerogels displayed the micron sized self-assembled tubular structures, which are highly entangled (Figure 1e,f). The self-assembly of caffeine and other pharmaceutical compounds into a tubular structure have been well studied in the literature. This prompted us to further investigate the self-assembly in detail. The diameter of the microcrystalline structures varied from 2 to 4 µm in diameter. Higher order structures do allow more detailed observation thereby providing evidence for an existing hypothesis that the gelation is due to self-assembled 3D network formation. Studying the native gel is a challenge, especially when the nanoscale structures are formed. On the other hand, optical microscopy has been used to directly visualize SAFIN formation, as well as their collapse [44,45,46,47,48]. Since, the caffeine fibers are relatively large it provides an opportunity to visualize gelation in its native state, where the network formation, its growth and entanglements can be followed in its native state.

**Figure 2:**
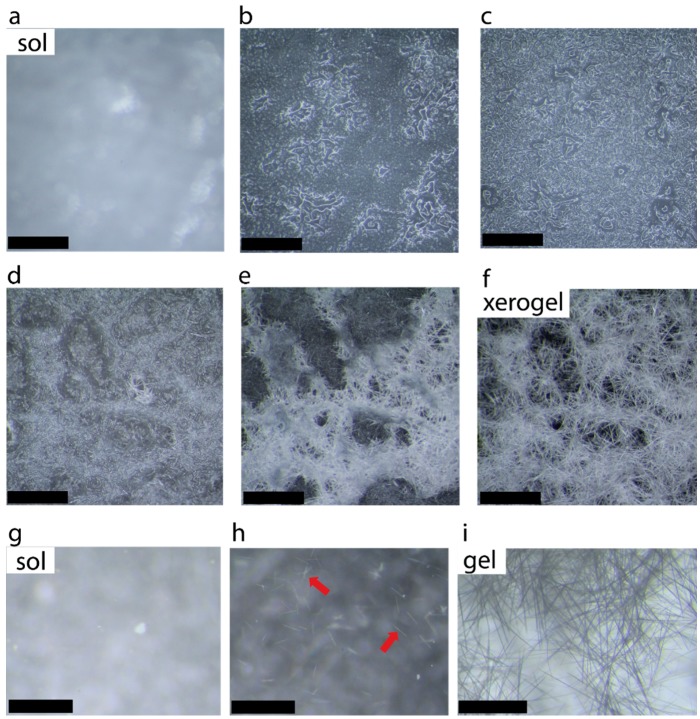
Photographs of 2.0% caffeine in 1-propanol (**a**–**f**) when a hot solution was placed on a glass slide (scale bar 0.85 mm) and (**g**–**i**) selected images of 2-0% caffeine in 1-propanol hot solution placed in a petri dish showing fiber formation in the presence of solvent (scale bar: 0.92 mm). Red arrows indicate the formatin of microcrystals.

The formation of microcrystalline network following by solvent immobilization is shown in Figure 1d. Figure 2a–f shows the selected photographs illustrating the growth and entanglement of microcrystalline network upon placing a hot solution of caffeine (2.0%) in 1-propanol on a glass slide. Figure 2g–i shows selected photographs of a hot solution in a Petri dish forming small crystallites, which nucleate the growth of entangled network in the presence of solvent.

**Figure 3:**
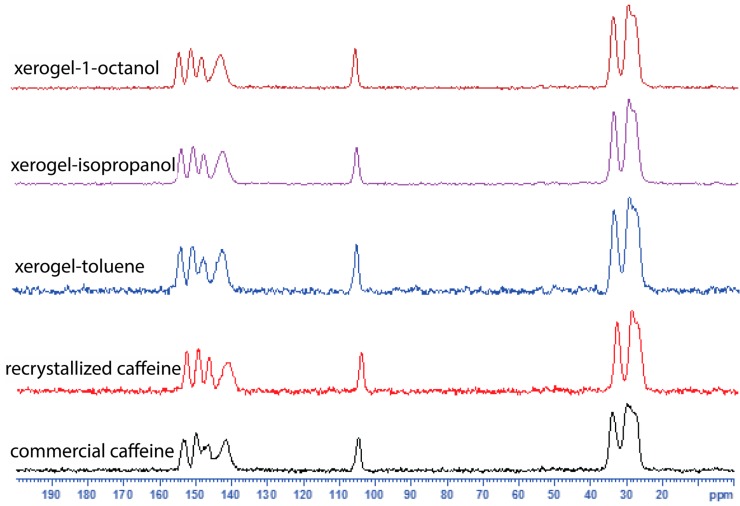
Solid state ^13^C CPMAS NMR spectra of commercial caffeine, recrystallized form and xerogels.

**Figure 4:**
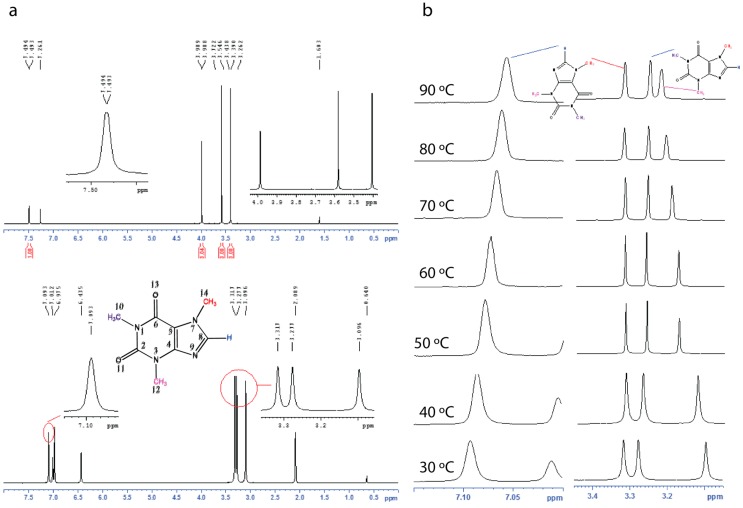
Solution NMR Studies; (**a**) ^1^H NMR of caffeine in CDCl_3_ (top) and 2.0% toluene-*d*_8_ gel of caffeine and (**b**) variable temperature (VT) NMR of 2.0% toluene-*d*_8_ gel of caffeine. Also see supplementary Figures S6–S8.

In order to compare the commercial caffeine, the recrystallized form and the xerogels, we used solid state ^13^C CPMAS NMR spectroscopy (Figure 3, see Supplementary Figure S5). The ^13^C CPMAS NMR results suggest that the recrystallized form of caffeine, and the xerogels display similar patterns in their ^13^C spectra and are comparable to the ^13^C CPMAS spectral patterns reported for the high temperature anhydrous form reported by Enright *et al.* [25]. The commercial caffeine, also shows a similar spectral pattern but the presence of overlapping peaks between 145–155 ppm and 25–30 ppm clearly suggests the presence of more than one form (Figure 3). We further confirm that both the commercial, and the recrystallized caffeine are anhydrous form of caffeine by elemental analysis. Finally, the gels were studied using solution ^1^H NMR (see Supplementary Figure S6) and variable temperature (VT) NMR spectroscopy (see Supplementary Figures S7–S10). The ^1^H NMR of the toluene-*d*_8_ gel showed broad signals, apart from that the signals from 10-CH_3_ and 12-CH_3_ now moved downfield (shielded) compared to that in CDCl_3_ solution (Figure 4). Upon heating both 10-CH_3_ and 12-CH_3_ signals showed a significant shift and while the 10-CH_3_ signal moved upfield upon heating, the 12-CH_3_ signal moved in the opposite direction. Meanwhile 8-H also shifted upfield in the NMR spectrum. Further, the variable temperature (VT) NMR of 1-octanol (non-deuterated) gel was performed (without field lock) to study the gel in its native state and similar observations were made (see Supplementary Figures S9–S11).

## 3. Conclusions

In summary, we have shown that anhydrous caffeine has the ability to act as a low molecular weight gelator, a property which has not been reported earlier. We provide direct evidence for microcrystalline fiber formation leading to entangled 3D network leading to gelation. Caffeine being naturally abundant, commercially cheap and one of the highly consumed alkaloids with medicinal properties, we foresee that this property is a significant finding to generate both materials and pharmaceutically important gels in combination with suitable components. Further studies to use caffeine as a two-component gelator towards hydrogels are progress in our laboratory, and the results will be published elsewhere.

## 4. Experimental Section

### 4.1. Materials and Methods

Analytical grade reagents and solvents were used for the purification, crystallization and gelation studies. The solvents were dried and stored over 3 Å molecular sieves prior to use. Caffeine was obtained from Aldrich (99%). All the solvents, used for recrystallization and gelation tests were purchased from commercial suppliers (Sigma Aldrich, Helsinki, Finland), methanol, ethanol, 1-propanol, *iso*-propanol, 1-butanol, 1-pentanol, 1-hexanol, 1-heptanol, 1-octanol, 1-nonanol, 1-decanol, cyclohexanol, ethylene glycol, 1,2-pentanediol, toluene, benzene, xylenes, mesitylene, chlorobenzene and benzyl alcohol. ^1^H and ^13^C NMR experiments were measured in a Bruker Avance DRX 500 NMR spectrometer equipped with a 5 mm diameter broad-band inverse probe head working at 500.13 MHz for ^1^H and at 125.76 MHz for ^13^C. The ^13^C{^1^H} NMR spectra were measured using composite pulse, waltz16, decoupling. The solution state NMR spectra were measured in CDCl_3_ and toluene-*d*_8_. ^1^H NMR of 1-octanol gel was performed without lock. ^1^H and ^13^C chemical shifts were referenced to the solvent signals (δ = 7.26 for CDCl_3_, and δ = 2.09 for toluene-*d*_8_) for ^1^H and δ = 77.0 ppm for ^13^C from int. TMS (Helsinki, Finland). Elemental analyses experiments were carried out using Elementar Vario EL III-analyser (Hanau, Germany).

### 4.2. Recrystallization of Caffeine

In a 250 mL round-bottom flask, a mixture of commercial caffeine (200 mg) and ethanol (25 mL) was heated over an oil bath until the mixture turned clear solution. The hot solution was quickly filtered using Whatman No. 4 filter paper (VWR International Oy, Helsinki, Finland) and allowed to crystallize. The crystalline solid was dried under vacuum and used for further studies.

### 4.3. Gelation Tests

A typical gelation test was performed dissolving a known amount of sample in a test tube (*l* = 10 cm, *d* = 1.0 cm) in a solvent (0.5 mL) under investigation. The mixture was heated slowly until it turns into a clear solution (note: We avoid rapid heating due to sublimation properties of caffeine, which may lead to erroneous results). The solution was either allowed to attain room temperature (~22 °C) or subjected for sonication for 1 min. The formation of the gel was tested using the “resistance to flow upon inversion of the test tube”. Depending on the appearance the gels are denoted as, P = precipitate after attaining a hot solution to room temperature. G = gel, Gs = required sonication, S = solution.

### 4.4. Scanning Electron Microscopy (SEM) Studies

The sample preparation for SEM measurements was carried out by placing the hot sol (10 µL) on a carbon tape fixed over a sample stub. The sample was allowed to dry under ambient conditions for 24 h. The sample was sputter coated with gold in a JEOL Fine Coat Ion Sputter JFC-1100 (Tokyo, Japan) and the images were collected using Bruker Quantax400 EDS microscope (Berlin, Germany) equipped with a digital camera.

### 4.5. Solid-State NMR Studies

The ^13^C{^1^H}CP/MAS and ^15^N{^1^H}CP/MAS NMR spectra were recorded on a Bruker AV400 spectrometer (Bremen, Germany) equipped with a 4 mm standard bore CPMAS probe head whose X channel was tuned to 100.62 MHz for ^13^C and 40.55 MHz for ^15^N, respectively. The other channel was tuned to 400.13 MHz for broad band ^1^H decoupling. Approximately 100 mg of dried and finely powdered samples were packed in the ZrO_2_ rotor closed with Kel-F cap and spun at 10 KHz rate. The ^13^C{^1^H}CPMAS NMR was carried out for all samples under Hartmann-Hahn conditions with TPPM (tppm15) decoupling. The π/2 pulse for proton and carbons were found to be 4.0 μs and 5 μs at power levels of −5.0 dB and −4.0 dB, respectively. The experiments were conducted at contact time of 2 ms. A total of 20,000 scans were recorded with 5 s recycle delay for each sample. All free inducation decays (FIDs) were processed by exponential apodization function with line broadening of 20 Hz prior to FT. The ^15^N{^1^H}CP/MAS NMR experiments were carried out for all samples at a 10 kHz spinning rate under Hartmann-Hahn condition. The π/2pulses for proton and nitrogen were found to be 4.2 µs and 5 µs at power levels of −4.6 dB and −0.8 dB, respectively. The optimized contact time of 2 ms was used for efficient polarization transfer with a 5 s recycle delay to acquire the CP/MAS spectra. A total of 50,000 scans were acquired to obtain the CP/MAS spectra.

### 4.6. Variable Temperature NMR of Toulene-d_8_ Gel

Twelve milligrams of recrystallized caffeine was taken in NMR tube (*d* = 5 mm) and 600 µL of tolune-*d_8_* was added. The sample was slowly heated until it turned into a clear solution. The solution was subjected for sonication and stabilized for 2 h. VTNMR was recorded from 30 °C to 90 °C with 10 °C increment at a time with 5 min of stabilizing time at each temperature.

### 4.7. Variable Temperature NMR of 1-Octanol Gel

Twelve milligrams of recrystallized caffeine was taken in NMR tube (*d* = 5 mm) and 600 µL of 1-octanol was added. The sample was slowly heated until it turned into a clear solution. The solution was subjected for sonication and stabilized for 2 h. VTNMR was recorded without lock from 30 °C to 90 °C with 10 °C increment at a time with 5 min of stabilizing time at each temperature.

### 4.8. Elemental Analysis

The elemental analyses were performed for the commercial and recrystallized form of caffeine using Elementar Vario EL III-analyser.

Elemental analysis of commercial caffeine: C, 49.31; H, 5.20; N, 28.62.
Elemental analysis of recrystallized form of caffeine: C, 49.66; H, 5.26; N, 28.78.
Theoretical composition of caffeine (C_8_H_10_N_4_O_2_): C, 49.48; H, 5.19; N, 28.85.

## Supplementary Materials

The following are available online at www.mdpi.com/2310-2861/2/1/9/s1, details of general experimental methods, recrystallization procedure, gelation tests, solid state NMR, electron microscopy and variable temperature NMR. Figures S1–S11; Table S1.
