# Supplementary material for: Caffeine as a Gelator
Source: Gels. 2016 Mar 2;2(1):9. doi: 10.3390/gels2010009 (PMC6318762; doi:10.3390/gels2010009)
Supplement: Supplementary file 1 [file gels-02-00009-s001.pdf]

Supplementary Materials: Caffeine as a Gelator

Nonappa and Erkki Kolehmainen

1. Experimental Section

Analytical grade reagents and solvents were used for the purification, crystallization and gelation studies. The solvents were dried and stored over 3 Å molecular sieves prior to use. Caffeine was obtained from Aldrich (99%). All the solvents, used for recrystallization and gelation tests were purchased from commercial suppliers (Aldrich), methanol, ethanol, 1-propanol, iso-propanol, 1-butanol, 1-pentanol, 1-hexanol, 1-heptanol, 1-octanol, 1-nonanol, 1-decanol, cyclohexanol, ethylene glycol, 1,2-pentanediol, toluene, benzene, xylenes, mesitylene, chlorobenzene and benzyl alcohol. ^1H and ^{13}C NMR experiments were measured in a Bruker Avance DRX 500 NMR spectrometer equipped with a 5 mm diameter broad-band inverse probe head working at 500.13 MHz for ^1H and at 125.76 MHz for ^{13}C. The $^{13}C\{^1H\}$ NMR spectra were measured using composite pulse, waltz16, decoupling. The solution state NMR spectra were measured in CDC_{13} and toluene-d_8. ^1H NMR of 1-octanol gel was performed without lock. ^1H and ^{13}C chemical shifts were referenced to the solvent signals ($\delta = 7.26$ for CDCl3, and $\delta = 2.09$ for toluene-d_8) for ^1H and $\delta = 77.0$ ppm for ^{13}C from int. TMS).

2. Recrystallization of Caffeine

In a 250 mL round-bottom flask a mixture of commercial caffeine (200 mg) and ethanol (25 mL) was heated over an oil bath until the mixture turned clear solution. The hot solution was quickly filtered using Whatman No. 4 filter paper and allowed to crystallize. The crystalline solid was dried under vacuum and used for further studies.

3. Gelation Tests

A typical gelation test was performed dissolving a known amount of sample in a test tube (l = 10 cm, d = 1.0 cm) in a solvent (0.5 mL) under investigation. The mixture was heated slowly until it turns into a clear solution (Note: We avoid rapid heating due to sublimation properties of caffeine, which may lead to erroneous results). The solution was either allowed to attain room temperature (~22 °C) or subjected for sonication for 1 min. The formation of the gel was tested using the "resistance to flow upon inversion of the test tube). Depending on the appearance the gels are denoted as, P = precipitate after attaining a hot solution to room temperature. G = gel, Gs = required sonication, S = solution (Table S1).

4. Scanning Electron Microscopy (SEM) Studies

The sample preparation for SEM measurements was carried out by placing the hot sol (10 μL) on a carbon tape fixed over a sample stub. The sample was allowed to dry under ambient conditions for 24 h. The sample was sputter coated with gold in a JEOL Fine Coat Ion Sputter JFC-1100 and the images were collected using Bruker Quantax400 EDS microscope equipped with a digital camera. The scanning electron microscopy images are shown in Figures S1 and S2.

5. Gel Melting Temperature (Tgel) Measurement

The gel melting temepratures were measured for different solvents (Figure S3) as well as for 1-octanol gel at different concentrations (Figure S4) using inverted test tube method.

6. Solid-State NMR Studies

The $^{13}C\{^1H\}$CP/MAS and $^{15}N\{^1H\}$CP/MAS NMR spectra were recorded on a Bruker AV400 spectrometer equipped with a 4 mm standard bore CPMAS probe head whose X channel was tuned to 100.62 MHz for ^{13}C and 40.55 MHz for ^{15}N, respectively. The other channel was tuned to 400.13 MHz for broad band ^1H decoupling. Approximately, 100 mg of dried and finely powdered

samples were packed in the ZrO_2 rotor closed with Kel-F cap and spun at 10 KHz rate. The $^{13}C\{^1H\}$CPMAS NMR was carried out for all samples under Hartmann-Hahn conditions with TPPM (tppm15) decoupling. The π/2 pulse for proton and carbons were found to be 4.0 μs and 5 μs at power levels of −5.0 dB and −4.0 dB, respectively. The experiments were conducted at contact time of 2 ms. A total of 20,000 scans were recorded with 5 s recycle delay for each sample. All FIDs were processed by exponential apodization function with line broadening of 20 Hz prior to FT (Figure S5).

7. Gelation Tests in Alcoholic and Aromatic Solvents

The results of gelation tests carried out using alcoholic and aromatic solvents are summarized below in Table S1.

Table S1. Gelation studies of caffeine. P= precipitate after attaining a hot solution to room temperature. G = gel, Gs = required sonication, S = solution.

Solvent	1.0%	1.5%	2.0%
Methanol	P	P	P
Ethanol	G^s	G^s	G
1-Propanol	G^s	G^s	G
1-Butanol	G^s	G^s	G
1-Pentanol	G^s	G^s	G
1-Hexanol	G^s	G^s	G
1-Heptanol	G^s	G^s	G
1-Otcanol	G^s	G^s	G
1-Nonanol	G^s	G^s	G
1-Decanol	G^s	G^s	G
Cycohexanol	G^s	G^s	G
t-Butanol	G^s	G^s	G
2-Pentanol	G^s	G^s	G
1,5-Pentanediol	G^s	G^s	G
4-heptanol	G^s	G^s	G
Cholorobenzene	P	G^s	G^s
1,2-Dichlorobenzene	P	G^s	G^s
Benzene	G^s	G^s	G^s
Toluene	G^s	G^s	G^s
Xylene	G^s	G^s	G^s
Mesitylene	G^s	G^s	G^s
Dichloromethane	S	S	S
Dioxan	S	S	S

8. Variable Temperature NMR of Toulene-d_8 Gel

Twelve milligrams of recrystallized caffeine was taken in NMR tube (d = 5 mm) and 600 μL of toluene-d_8 were added. The sample was slowly heated until it turned into a clear solution. The solution was subjected for sonication and stabilized for 2 h. VTNMR was recorded from 30 °C to 90 °C with 10 °C increment at a time with 5 min of stabilizing time at each temperature (Figures S6–S8).

9. Variable Temperature NMR of 1-octanol Gel and Tgel Measurement

Twelve milligrams of recrystallized caffeine was taken in NMR tube (*d* = 5 mm) and 600 μL of 1-octanol were added. The sample was slowly heated until it turned into a clear solution. The solution was subjected for sonication and stabilized for 2 h. VTNMR was recorded without lock from 30°C to 90 °C with 10 °C increment at a time with 5 min of stabilizing time at each temperature (see Figures S9–S11).

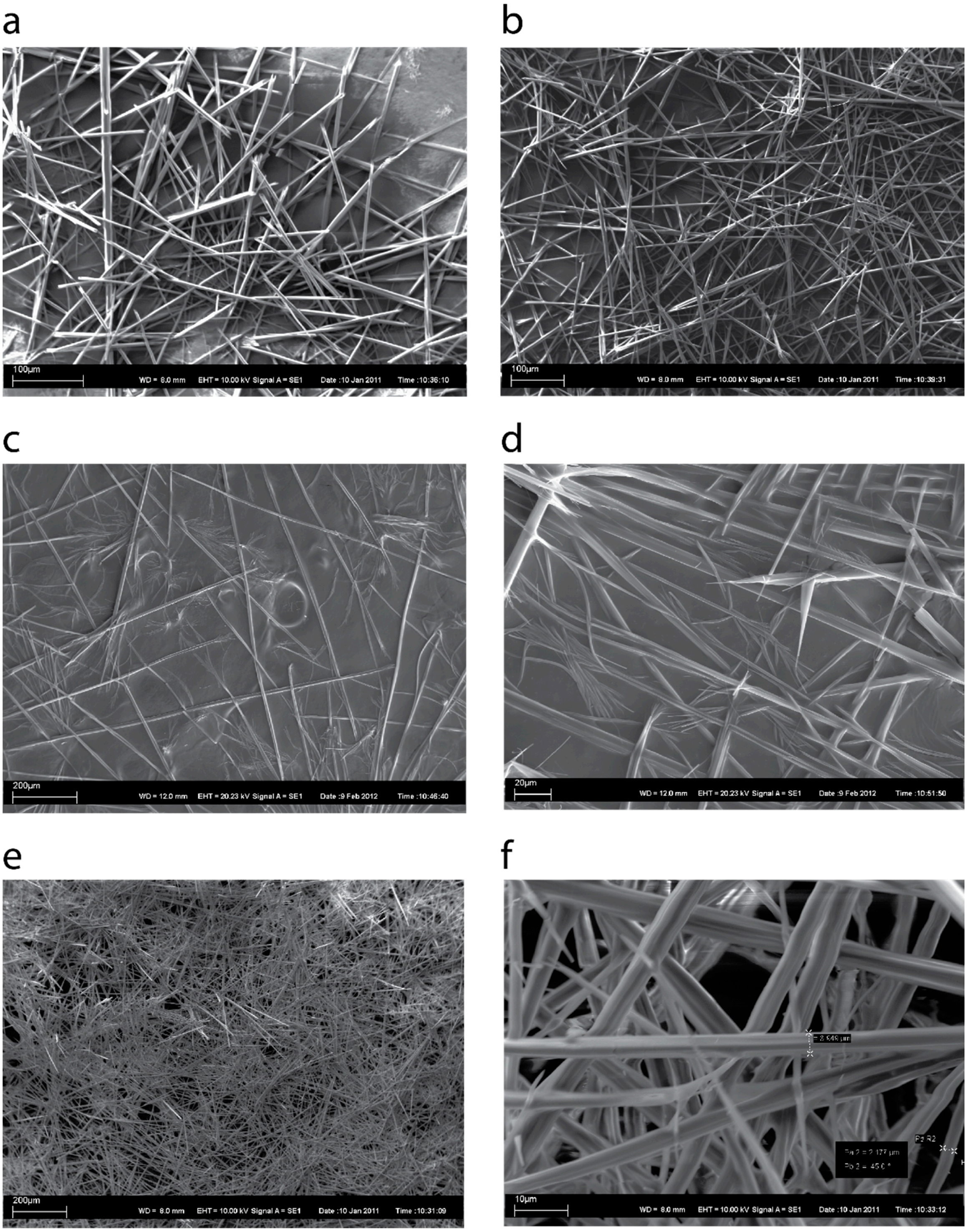

Figure S1. Scanning Electron Micrographs of xerogels obtained from 2.0% gels from (**a**) ethanol; (**b**) 1-propanol; (**c**) 1-butanol; (**d**) 1-pentanol; (**e**) and (**f**) 1-octanol.

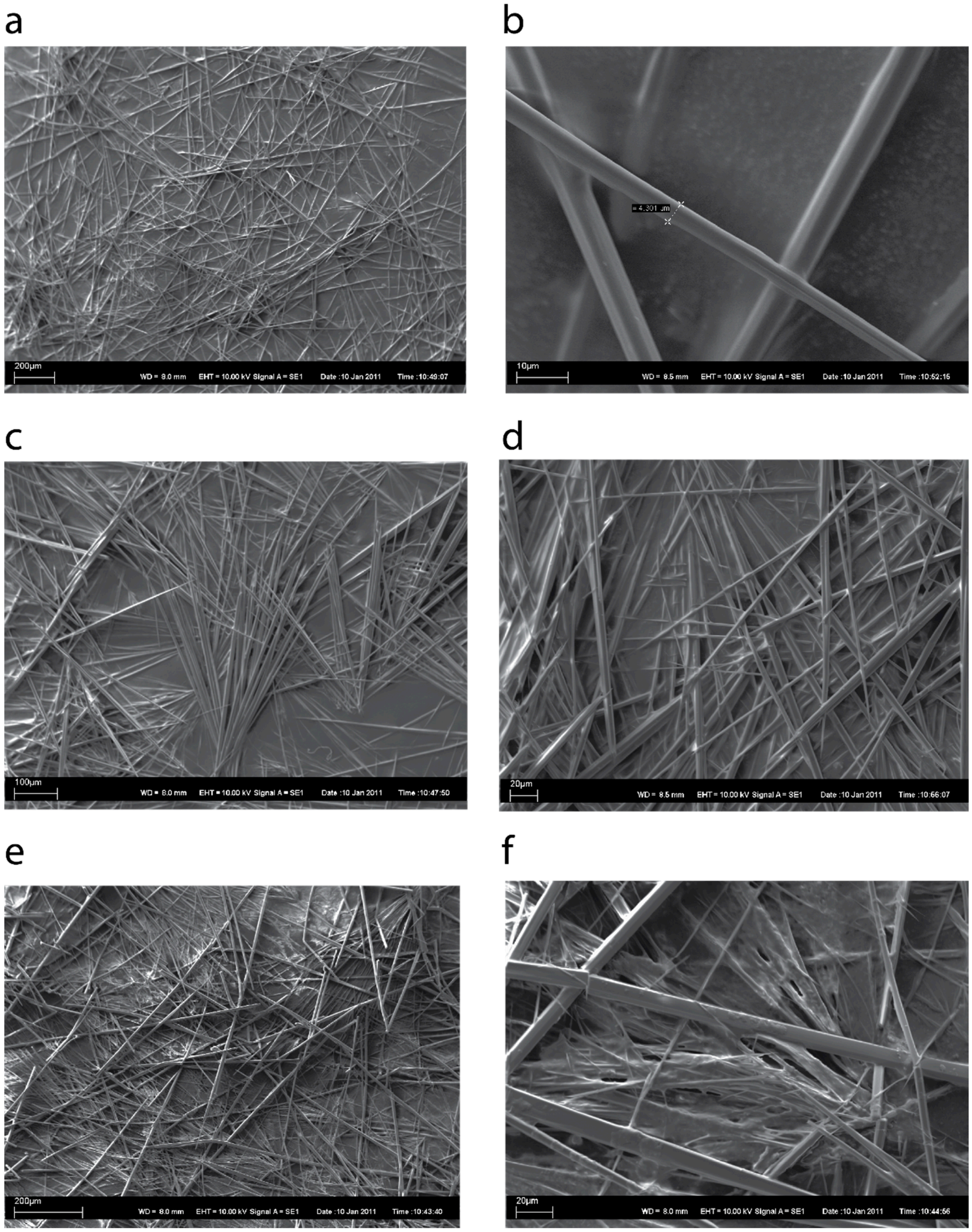

Figure S2. Scanning electron micrographs of xerogels obtained from 1.0% (caffeine) gels from (**a**) and (**b**) toluene; (**c**) m-xylene; (**d**) mesitylene; (**e**) and (**f**) chlorobenzene

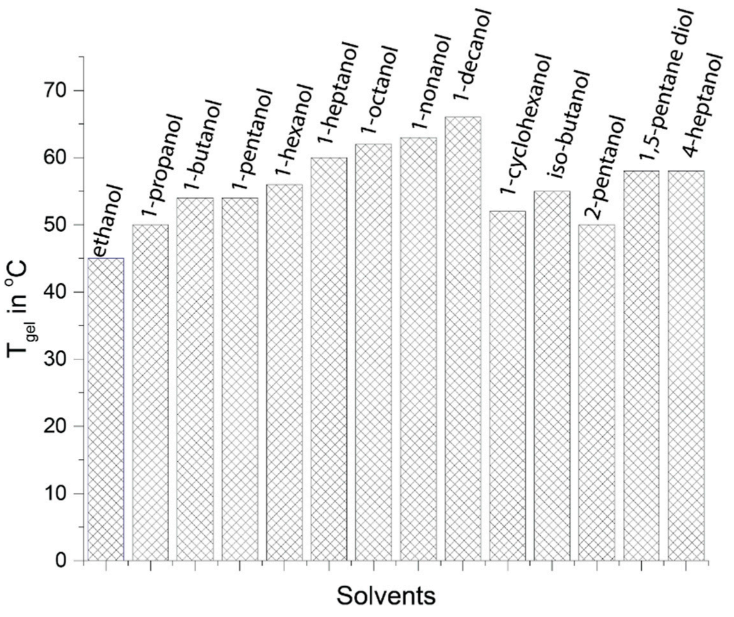

Figure S3. Gel melting temperature of 2.0% caffeine in different solvents. Heating rate 2.5 °C.

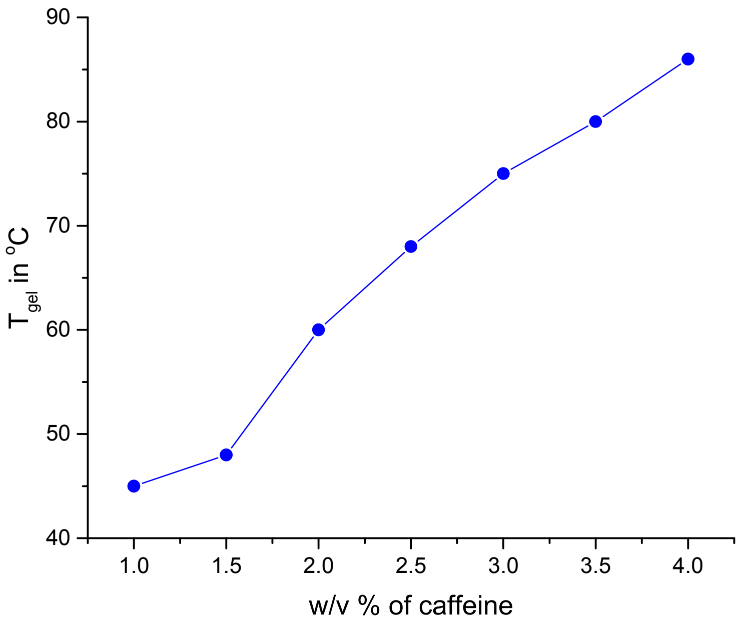

Figure S4. Tgel of 1-Octanol gel derived from caffeine as a function of concentration.

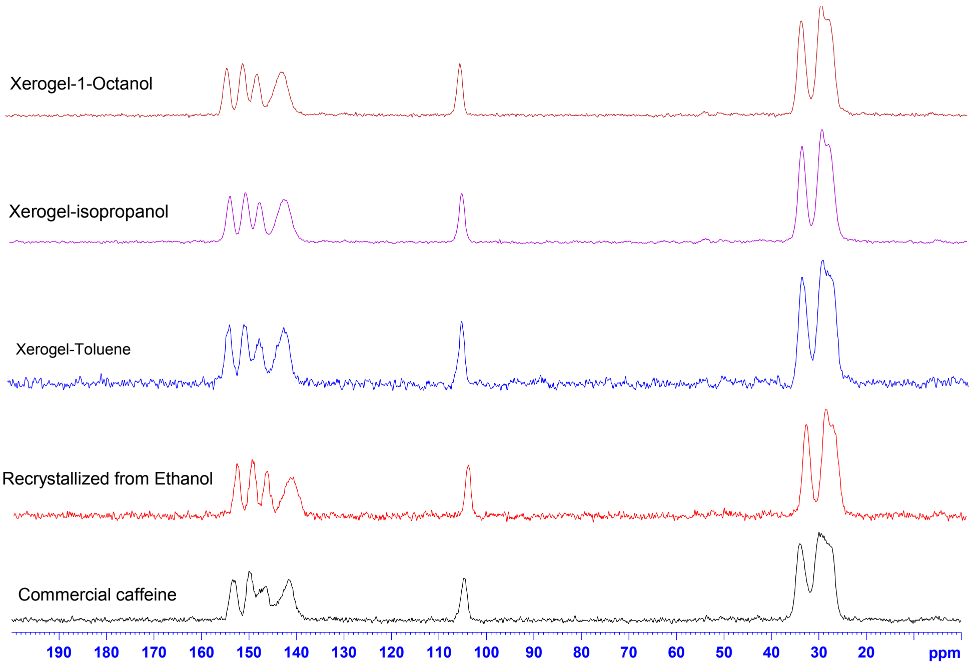

Figure S5. ^{13}C CPMAS NMR spectra of commercial, recrystallized sample and xerogels.

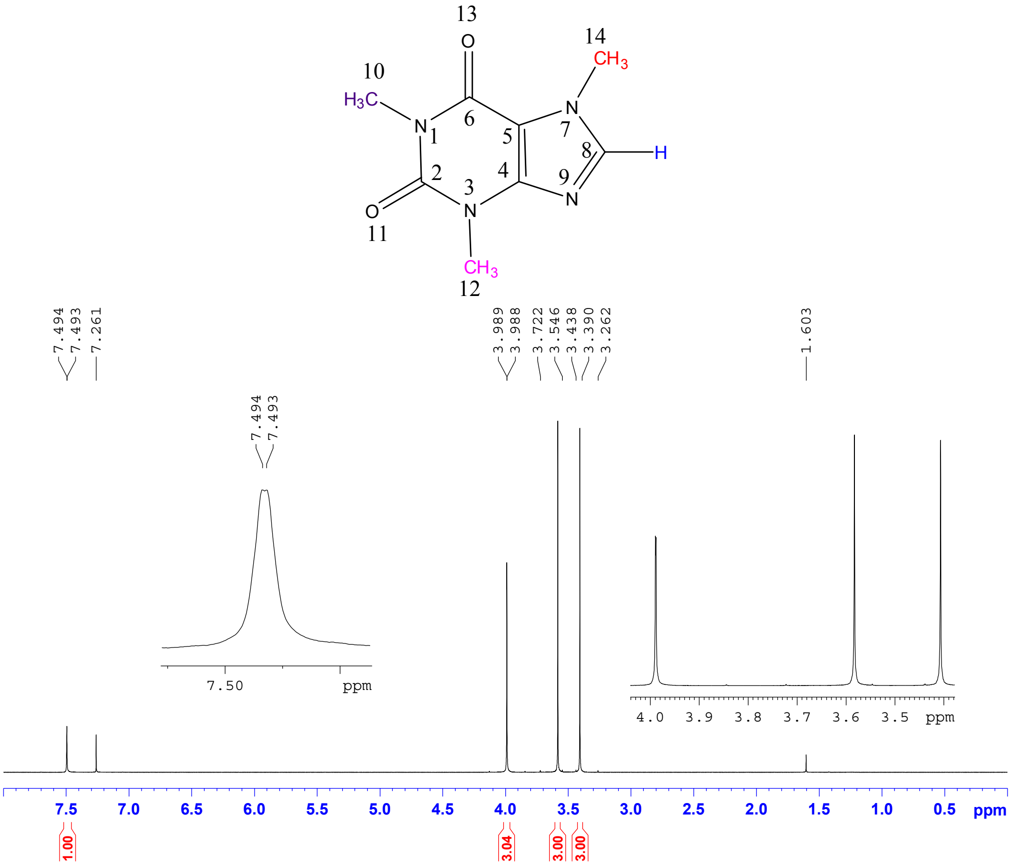

Figure S6. ^{1}H NMR spectra of caffeine in $CDCl_3$.

The ^{1}H NMR of caffeine in $CDCl_3$ (clear solution) shows the vinylic porton (C-8 protons) having 4 bond coupling with C-14 protons (CH_3) and appears as a broad quartet (Figure S6). The proton signals from N-CH_3´s are well separated from each other.

The ^{1}H NMR of the toulene-d_8 gel (Figure S7) showed broad signals, apart form that the signals from 10-CH_3 and 12-CH_3 now moved downfield (Shielded) compared to that in $CDCl_3$ solution. Upon

heating both 10-CH_3 and 12-CH_3 signals showed a significant shift and while 10-CH_3 signal moved upfield upon heating, the 12-CH_3 signal moved in the opposite direction. Meanwhile 8-H also shifted upfield in the nmr spectrum (Figures S8–S11). This observation is attributed to involvement of carbonyl oxygens O11 and O13 and N-9 in hydrogen bonding.

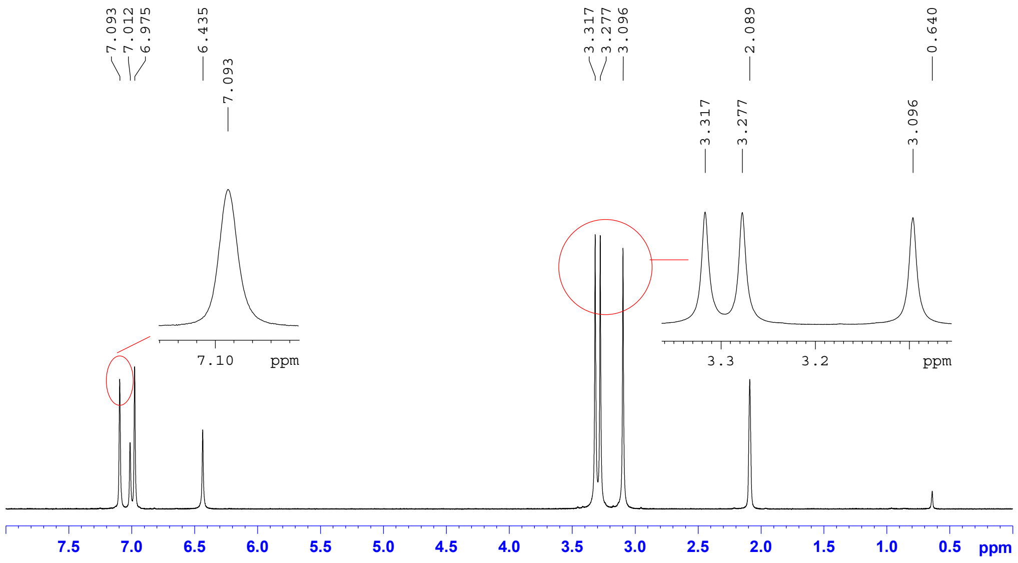

Figure S7. ^{1}H NMR spectrum of toluene-d_8 gel (2%) w/v.

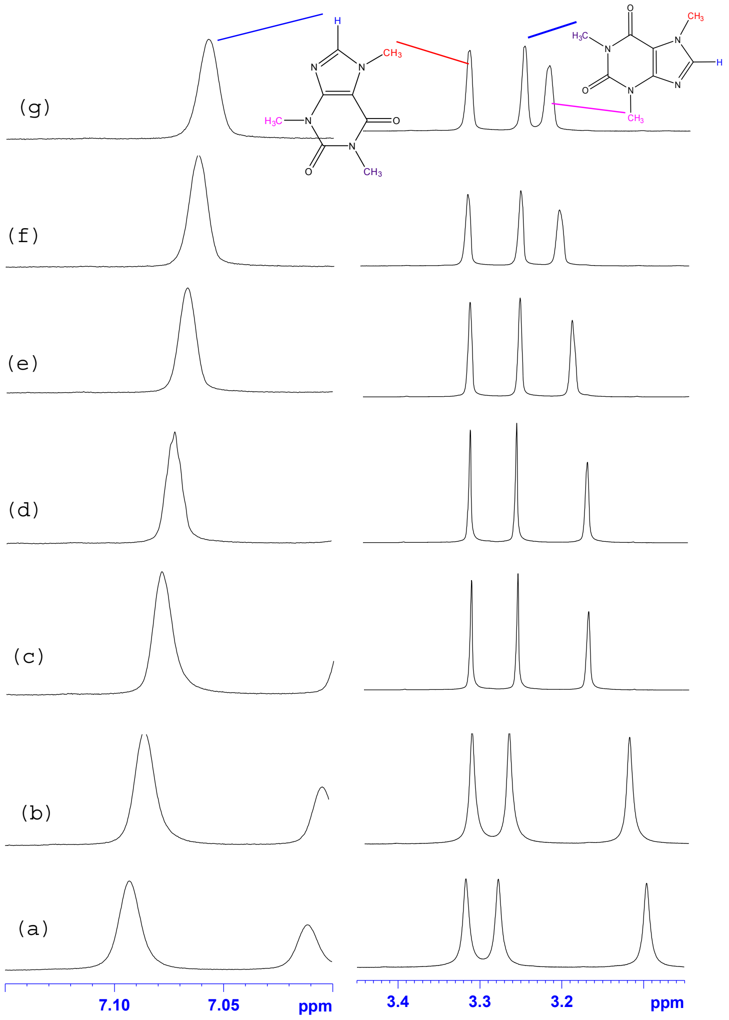

Figure S8. VT NMR spectra of toluene-d_8 gel (**a**) 30 °C; (**b**) 40 °C; (**c**) 50 °C; (**d**) 60 °C; (**e**) 70 °C; (**f**) 80 °C and (**g**) 90 °C.

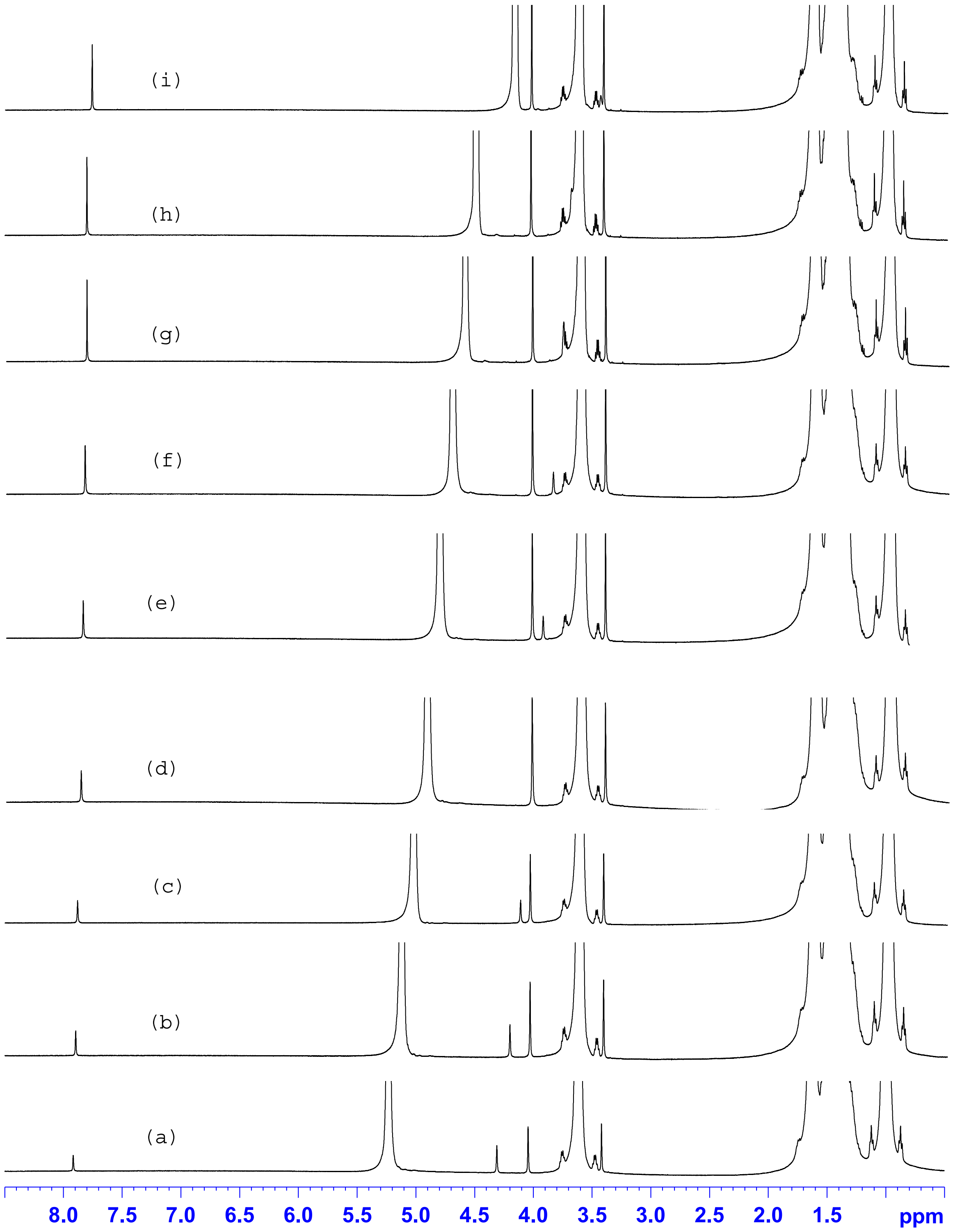

Figure S9. VT NMR spectra of 1-Octanol gel of caffeine (**a**) 30 °C; (**b**) 35 °C; (**c**) 40 °C; (**d**) 45 °C; (**e**) 50 °C; (**f**) 55 °C; (**g**) 60 °C; (**h**) 65 °C; (**i**) 70 °C.

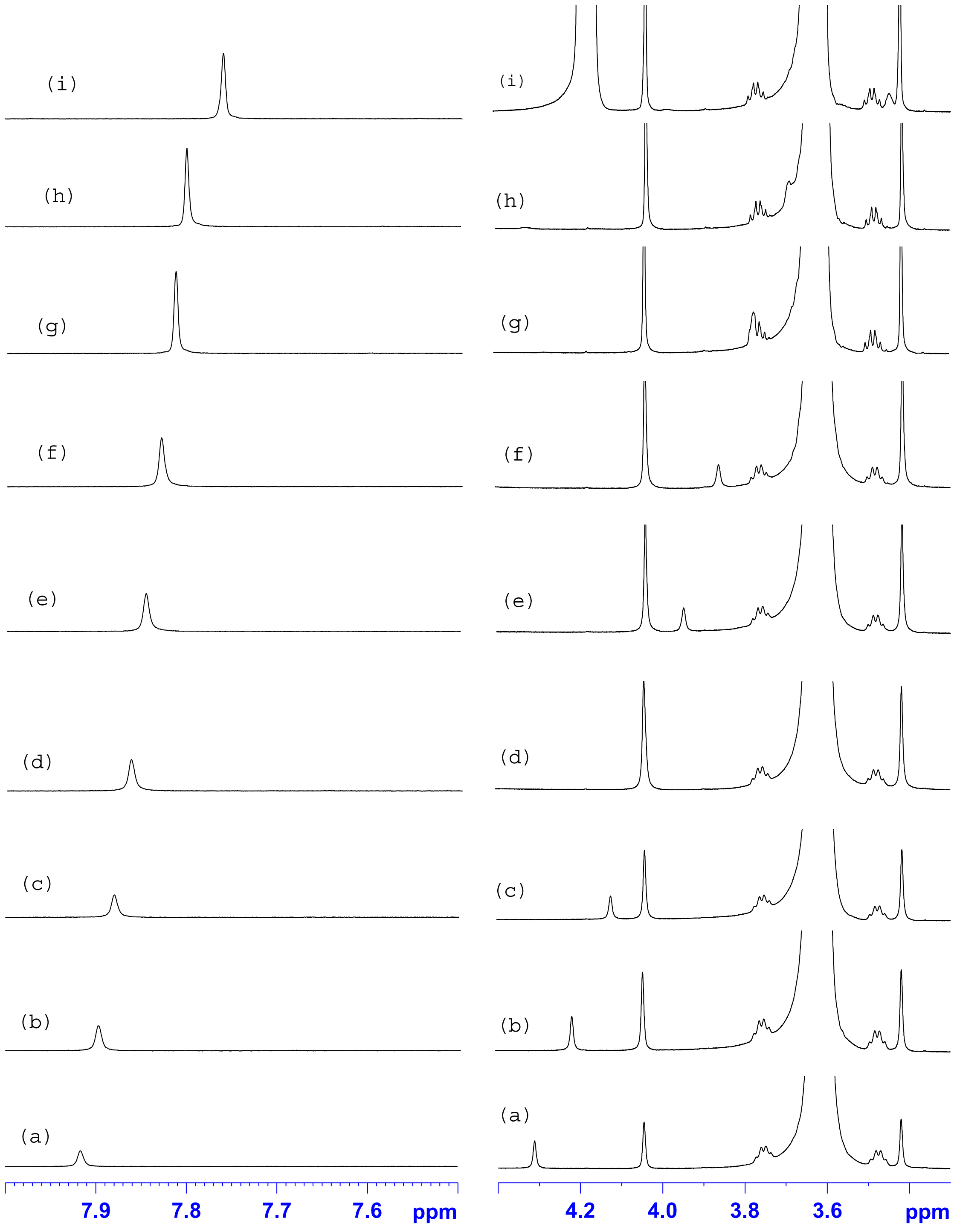

Figure S10. VT NMR spectra of 1-octanol gel of caffeine (**a**) 30 °C; (**b**) 35 °C; (**c**) 40 °C; (**d**) 45 °C; (**e**) 50 °C; (**f**) 55 °C; (**g**) 60 °C; (**h**) 65 °C; (**i**) 70 °C.

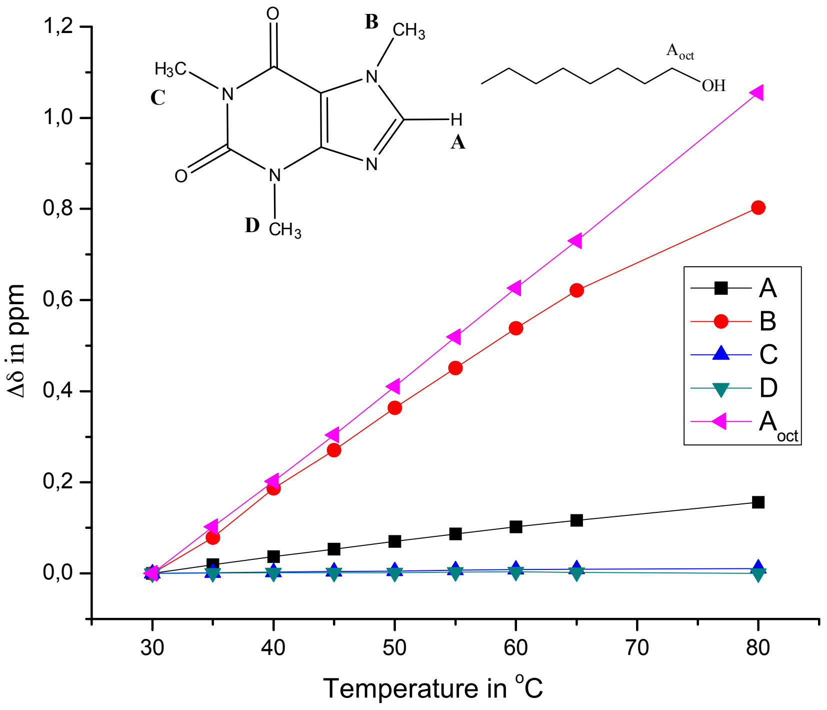

Figure S11. Relative change in the chemical shift values of protons of caffeine and 1-octanol with change in the temperature of 2% 1-octanol gel of caffeine.

© 2016 by the authors; licensee MDPI, Basel, Switzerland. This article is an open access article distributed under the terms and conditions of the Creative Commons by Attribution (CC-BY) license (http://creativecommons.org/licenses/by/4.0/).
